# Gone with the plate: the opening of the Western Mediterranean basin drove the diversification of ground-dweller spiders

**DOI:** 10.1186/1471-2148-11-317

**Published:** 2011-10-31

**Authors:** Leticia Bidegaray-Batista, Miquel A Arnedo

**Affiliations:** 1 Institut de Recerca de la Biodiversitat & Departament de Biologia Animal, Universitat de Barcelona, Av. Diagonal 643, 08020, Barcelona, Spain

## Abstract

**Background:**

The major islands of the Western Mediterranean--Corsica, Sardinia, and the Balearic Islands--are continental terrenes that drifted towards their present day location following a retreat from their original position on the eastern Iberian Peninsula about 30 million years ago. Several studies have taken advantage of this well-dated geological scenario to calibrate molecular rates in species for which distributions seemed to match this tectonic event. Nevertheless, the use of external calibration points has revealed that most of the present-day fauna on these islands post-dated the opening of the western Mediterranean basin. In this study, we use sequence information of the *cox1*, *nad1*, *16S*, *L1*, and *12S *mitochondrial genes and the *18S*, *28S*, and *h3 *nuclear genes, along with relaxed clock models and a combination of biogeographic and fossil external calibration points, to test alternative historical scenarios of the evolutionary history of the ground-dweller spider genus *Parachtes *(Dysderidae), which is endemic to the region.

**Results:**

We analyse 49 specimens representing populations of most *Parachtes *species and close relatives. Our results reveal that both the sequence of species formation in *Parachtes *and the estimated divergence times match the geochronological sequence of separation of the main islands, suggesting that the diversification of the group was driven by Tertiary plate tectonics. In addition, the confirmation that *Parachtes *diversification matches well-dated geological events provides a model framework to infer substitution rates of molecular markers. Divergence rates estimates ranged from 3.5% My^-1 ^(*nad1*) to 0.12% My^-1 ^(*28S*), and the average divergence rate for the mitochondrial genes was 2.25% My^-1^, very close to the "standard" arthropod mitochondrial rate (2.3% My^-1^).

**Conclusions:**

Our study provides the first unequivocal evidence of terrestrial endemic fauna of the major western Mediterranean islands, whose origin can be traced back to the Oligocene separation of these islands from the continent. Moreover, our study provides useful information on the divergence rate estimates of the most commonly used genes for phylogenetic inference in non-model arthropods.

## Background

The estimation of the timing of evolutionary events from DNA sequence information has become a major research topic in evolutionary biology. Although the use of molecular data to estimate divergence times goes back to the mid 60's of the past century [1], the number of studies that include time estimation has increased rapidly over the last decade due to the ever increasing amount of DNA sequence data and the development of new algorithms that relax the limiting assumptions of the molecular clock (see reviews of [2-4]). Information on timescales has shed light not only on the origin of taxonomic groups but has also allowed for the testing of biogeographic and climatic hypotheses, the estimation of rates of species diversification, and the investigation of rates of molecular evolution, among other topics [5].

Genetic distances are transformed into absolute divergence times by incorporating calibration points. The choice of a specific calibration date is therefore potentially crucial to accurately infer molecular dates (e.g., [6]). Biogeographic and paleoecological data are among the main sources of information for dating phylogenetic nodes, either as a complement to the fossil record, or as the only available evidence in poorly preserved organisms. The use of geologic and paleoclimatic evidence, however, has been criticised on the basis of their limited accuracy and unwarranted assumptions (e.g., [7-9]). Therefore, the appropriate use of biogeographic events to calibrate phylogenies requires a well-documented geochronology and the demonstration that the assumed barriers constitute a true obstacle to dispersal for the focal group.

**Figure 1:**
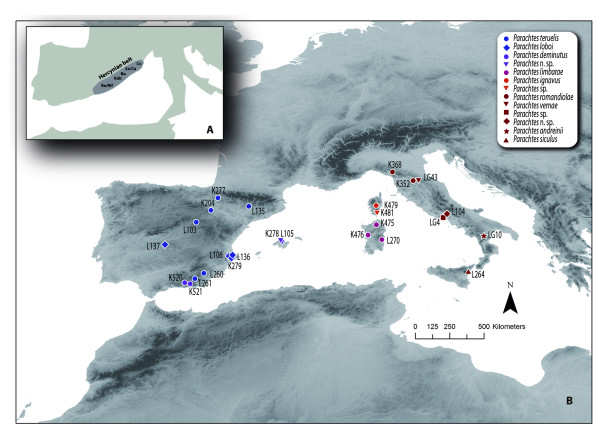
**Maps of the western Mediterranean Basin**. (A) Map showing the presumed location of the microplates that formed part of the Hercynian belt (modified from Rosenbaum et al.[12]). Microplate names are indicated as follow: Be/Rif corresponds to theBetic/Rift plate, Kab to the Kabylies, Ba to the Balearic Islands, Sa/Ca to Sardinia/Calabria and Co to Corsica. (B) Map showing sampling locations and codes of *Parachtes *specimens sampled. Species are indicated by symbols and colours (see legends in the upper right box); the colours correspond to the main lineages indicated in Figure 2 and Figure 3. See Additional file 1 for detailed information of sample codes.

The complex geological evolution of the western Mediterranean region, located at the edge of the converging African and Eurasian plates, is relatively well understood [10-17] and has been extensively used for molecular dating (e.g., [18-24]) as well as to explain present-day species distributions (e.g., [25]). The opening of the western Mediterranean basin started during the Alpine orogeny, when an eastward tectonic extension occurred between the African and Eurasian plates due to a subduction rollback between the oceanic and the continental slab (back arc extension). As a result, at the beginning of the Oligocene (~30-25 million years ago, Ma), several continental microplates that formed part of the Hercynian belt (Corsica, Sardinia, Balearic Islands, Calabro-Peloritan massif, including northern Sicily, the Kabylies and the Beatic-Rift Cordillera) broke off and started drifting from the eastern Iberian Peninsula and southern France to their present-day location (Figure 1(A)). During the first stage of the back arc extension, the northern microplate assemblage (Sardinia, Corsica and Calabro-Peloritan massif) started drifting counter-clockwise with respect to the Eurasian plate, whereas the southern assemblage (the Balearic Islands and Great Kabylie) drifted clockwise relative to Iberia. The movement of the northern microplates resulted in the opening of the Ligurian Sea, the Valencia Trough and the Gulf of Lyon. Corsica, Sardinia and the Calabro-Peloritan massif collided with the Apulian plate around 20-18 Ma, completing the formation of the Gulf of Lyon. There is no general consensus on the time of separation of Corsica and Sardinia, although it almost certainly occurred during the Corsica-Sardinia rotation. This rotation has been dated to approximately 21-15 Ma [13,26] based on paleomagnetic data and is further corroborated by the presence of early Burdigalian marine sediments in southern Corsica [27]. Likewise, there are doubts about the time when the exotic blocks of the Calabro-Peloritan massif drifted off from Sardinia to their present-day positions, although it is generally accepted that it occurred during the formation of the Tyrrhenian Sea [10,17]. According to Rosenbaum & Lister [28], the Tyrrhenian Sea started opening 10-9 Ma in the northern and western part, finishing around 5-4 Ma in the southern part. The same geological processes that generated the Tyrrhenian Sea also broke off the land connection between the Maghrebides and Sicily, opening the Strait of Sicily [12]. The Provençal and the Algerian basins are both older than the Tyrrhenian Sea, and originated during the Early-Middle Miocene (21-15 Ma), when the Kabylies block broke off from the Balearic Islands (~21 Ma) and started drifting southward until they collided with the African margin (18-15 Ma). The formation of the Alboran basin is a matter of debate because the original position of the Rif-Betic mountain range is uncertain [12,15]. Some authors suggest that during the Oligocene, the Rif-Betic mountain range formed a continuous orogenic belt together with the western Alps, Calabria, Corsica and the Kabylies blocks [12]. At the time of the main subduction rollback, the Rif-Betic block drifted southwest up to its present location (~10 Ma), completing the formation of the Alboran basin.

Although the formation of the main western Mediterranean islands has been used to date evolutionary divergence times and to explain species ranges, most studies using independent calibration points or substitution rates estimated from related organisms have concluded that the origin of present-day fauna in the region greatly post-dates the opening of the basin [29-33]. In particular, the Messinian Salinity Crisis (MSC) has been pointed out as one of the main drivers of local diversification. The late Miocene (8 Ma) marine corridors in southern Iberia and northern Morocco closed about 5.96 Ma, isolating the Mediterranean Sea from the Atlantic Ocean and causing a large reduction in Mediterranean water levels and the emergence of land connections between North Africa, Corsica, Sardinia and Eurasia and between the Balearic Islands and Iberia [34,35,16]. The opening of the Strait of Gibraltar about 5.3 Ma restored the water exchange between the Atlantic and Mediterranean waters, reestablishing effective isolation of the island ecosystems [36,37]. To date, the plant family Araceae constitutes the only case reported of Mediterranean organisms in which diversification has been shown to correspond to the Oligocene break-off of the Hercynian belt [38].

The ground-dweller spider genus *Parachtes *Alicata, 1964 (Araneae, Dysderidae) is a promising candidate for a terrestrial animal whose diversification may have been shaped by western Mediterranean plate tectonics. This genus is restricted to the western Mediterranean, where it exhibits a disjunct distribution [39]. It currently includes 12 species: *P. ignavus *Simon, 1882 and *P. inaequipes *Simon 1882 from Corsica, *P. limbarae *Kraus, 1955 from Sardinia, *P. siculus *Caporiacco, 1949 from Sicily, *P. romandiolae *Caporiacco, 1949, *P. vernae *Caporiacco, 1936, *P. latialis *Alicata, 1966 and *P. andreinii *Alicata, 1966 from the Italian Peninsula, and *P. cantabrarum *Simon, 1914, *P. teruelis *Kraus, 1955, *P. loboi *Jiménez-Valverde, 2006 and *P. deminutus *Denis, 1957 from the Iberian Peninsula. Two new species, one endemic to Majorca (Balearic Islands) and another to the Italian Peninsula (Lazio), have been recently discovered and are awaiting formal description (Arnedo, unpublished data). *Parachtes *shows a remarkable uniformity in its somatic morphology and most species' diagnostic features are restricted to the male and female genitalia [39]. *Parachtes *species are nocturnal wandering hunters, usually found in leaf-litter, under dead logs or stones and in dark and humid habitats at mid to high altitudes (> 500 m). These spiders do not build webs, and ballooning (i.e., aerial dispersal by means of silk threads) has been reported neither in the genus nor in the whole family. The low vagility and habitat preferences suggest limited opportunities for overseas dispersal, although transport by floating islands has been proposed to explain colonisation of the oceanic Canary Islands by the closely related genus *Dysdera *[40].

Here, we investigate the role of the major geological events associated with the opening of the Western Mediterranean basin in shaping diversification of the spider genus *Parachtes*, by inferring a molecular phylogeny based on eight mitochondrial and nuclear genes from a thorough taxonomic sampling of the genus and its close relatives. Relaxed clock models in combination with multiple, independent biogeographic and fossil calibration points are further used to reconstruct the temporal framework of species diversification.

## Results

### Samples and sequences analysed

The specimens and sequences analysed in the present study are summarised in Additional file 1. The alignment of the non-protein coding genes, including informative gap characters coded as absence/presence characters (see Additional file 2 for details), were merged with the protein-coding genes (*cox1 *= 1257, *nad1 *= 358 and *h3 *= 327 characters), resulting in a combined matrix of 4454 characters for the "default alignment", 4462 for the "gappy alignment" and 4447 for the "compressed alignment". Gap characters were included in the parsimony and Bayesian inference analyses but excluded in maximum likelihood and divergence time analyses.

### Phylogenetic analyses

**Figure 2:**
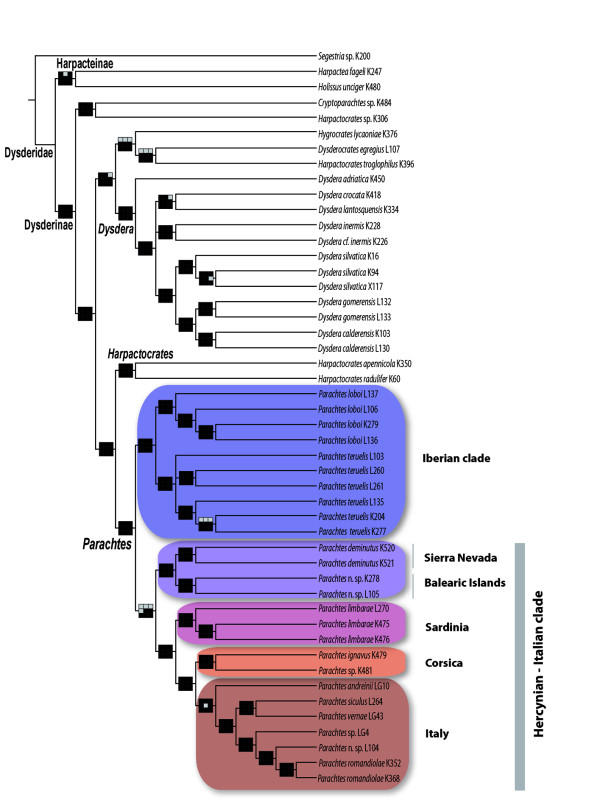
**Strict consensus of the six most parsimonious trees**. The most parsimonious tree (8458 steps, CI: 33, RI: 62) obtained from the default alignment, summarising parsimony, maximum likelihood and Bayesian inference supports. Clade support: black square, supported by Parsimony jackknife > 70%, Maximum likelihood bootstrap > 70%, and Bayesian posterior probability > 0.95; grey square, clade recovered but with support below the threshold value above; white squares, clade not recovered. Upper line: parsimony on default alignment (left), parsimony on gappy alignment (centre), parsimony on compressed alignment (right); middle line: Maximum likelihood on partition scheme P1 (left), P2 (centre), P3 (right); lower line: Bayesian inference on partition scheme P1 (left), P2 (centre), P3 (right).

Preliminary analysis using parsimony conducted on the concatenated matrices of the default, gappy and compressed alignments resulted in almost identical topologies and similar jackknife supports, although jackknife values were slightly higher for the default alignments (Figure 2). The *12S *and *16S-L1 *genes were the most variable and hence the most sensitive to changes in the alignment parameter values, recovering slightly different topologies. Differences, however, involved unsupported alternative positions.

Bayesian inference and maximum likelihood analyses were only conducted on the default alignment concatenated matrix, because no remarkable differences were found among the resulting topologies of each alignment. The selected substitution models for each gene and gene partition are shown in Additional file 3. Phylogenetic analyses under the three inference methods and partition strategies resulted in almost identical topologies, summarised in Figure 2.

All methods support the monophyly of *Parachtes *and its sister group relationship to the western Mediterranean species of *Harpactocrates*. As suspected [41], the eastern European species currently included in *Harpactocrates *did not form a clade with its western counterparts. The most basal split in the genus *Parachtes *separates species of the Iberian Peninsula (Iberian clade) from those of the western Mediterranean islands, Italy and the Betic species (Hercynian-Italian clade). Although all analyses support this basal split, the support for the Hercynian-Italian clade was moderate to low (parsimony jackknife 67%, Bayesian posterior probability 0.95, maximum likelihood bootstrap 51%), and the AU topology test could not reject the alternative topology where all Iberian and the Balearic species formed a clade sister to the remaining species (p = 0.464, 0. 469, 0.473, for P1, P2 and P3, respectively). Maximum likelihood and Bayesian inference analyses conducted on P2 and P3 partition schemes, however, increased support for the Hercynian-Italian clade (72/0.96 and 84/0.98, respectively, see Figure 2). The Hercynian-Italian clade was further resolved into two clades, one clade including the Balearic and Betic species, and a second clade where the Italian species form a monophyletic group sister to Corsica, with both of them in turn sister to Sardinia. All of the former clades were highly supported.

### Temporal framework of *Parachtes *diversification

Bayes factor analysis provided decisive support for selecting relaxed clock models against the strict clock, but could not discriminate between the two Bayesian relaxed clocks. The lognormal model, however, yielded the highest harmonic mean likelihood and was used in subsequent analyses. Likewise, BEAST analyses using Yule as tree prior yielded the highest likelihood harmonic means, but provided no strong evidence as compared to the more complex birth-death models. Finally, Bayes factors indicated decisive evidence in favour of the P3 partition scheme over the alternative schemes (Bayes factors summarised in Additional file 4).

**Figure 3:**
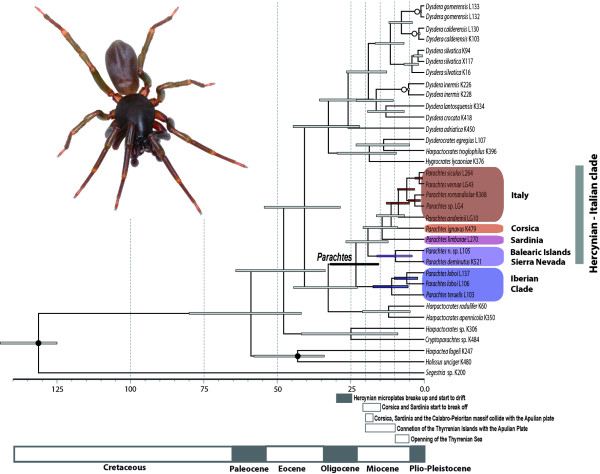
**Chronogram obtained with BEAST using fossil and biogeographic calibration points**. Chronogram obtained under partition scheme P3 (by genes, and protein coding genes 1^st ^+ 2^nd ^position vs. 3^rd ^position), Yule speciation tree prior model, and lognormal uncorrelated relaxed clock. The *x*-axis scale is in million years. Bars indicate 95% HPD intervals. Filled circles indicate fossil constraints and open circles indicate biogeographic constraints (see Material and Methods for details). The geological time scale and the main geological events that occurred during the opening of the western Mediterranean Basin are indicated below the *x*-axis. Photograph of a *Parachtes vernae *male (photo credits E. Mateos).

The chronogram with corresponding confidence intervals obtained with the preferred analysis options is shown in Figure 3. The time of the split between the Iberian clade and the Hercynian-Italian clade was estimated at 23.16 Ma (31.81-15.32 Ma), while the time of divergence between the Balearic-Betic clade and the Sardinian-Corsican-Italian clade was estimated at 19.17 Ma (26.63-12.24). The Baleares and the Betic clades split at 9.73 Ma (16.14-4.09). Sardinia split from the Corsican-Italian clade at 14.34 Ma (20.81-9.00), and the Corsican lineage split from the Italian clade at 11.27 Ma (16.28-6.65), similar to the estimated time of divergence of the two Iberian species *P. loboi *and *P. teruelis*, 11.09 Ma (17.39-5.50). The southern Italian species *P. andreinii *diverged from the remaining Italian species at 8.80 Ma (12.93-4.96). Italian *P. siculus *and *P. vernae *split from *P. romandiolae *and *P*. n. sp. at 5.90 Ma (9.14-3.23). The most recent species splits correspond to the two former pairs, at 3.20 Ma (5.40-1.26) and 1.69 Ma (3.14-0.56), respectively.

The substitution rates inferred for each gene partition were (mean pairwise divergence My^-1^): 3.5% (*nad1*), 2.5% (*cox1*), 1.7% (*12S*), 1.3% (*16S*-*L1*), 0.22% (*h3*) and 0.12% (*28S*) (Table 1). The *18S *substitution rate could not be estimated due to low genetic variability and limited phylogenetic resolution. All genes evolved in a non-clocklike manner, as indicated by ucld.stdev values and the high coefficient of variation, which reflects heterogeneity in substitution rates along tree branches. The nuclear gene 28S showed higher deviation from the strict clock, as ucld.stev and the coefficient of variation were greater than 1. Moreover, the 95% HPD interval of the covariance values span zero in all genes, which means that there is not evidence of autocorrelation of rates in the phylogeny (see BEAST user manual).

**Table 1:** Estimated substitution rates per gene obtained with BEAST based on fossil and biogeographic calibration points

Gene	Mean Rate	ucld.mean	ucld.stdev	Coefficient Variance	Covariance
***cox1***	0.0125 (0.0089-0.0165)	0.0199 (0.0136-0.0270)	0.6668 (0.4780-0.8728)	0.7087 (0.4823-0.9635)	0.0254 (-0.2254-0.2960)
***nad1***	0.0177 (0.0115-0.0240)	0.0258 (0.0163-0.0362)	0.6390 (0.4395-0.8792)	0.6400 (0.4088-0.8978)	0.0003 (-0.2474-0.2699)
***12S***	0.0087 (0.0057-0.0119)	0.0128 (0.0080-0.0181)	0.6950 (0.4080-1.0218)	0.7272 (0.3649-1.1102)	-0.0115 (-0.2637-0.2773)
***16-L1***	0.0063 (0.0046-0.0082)	0.0091 (0.0063-0.0124)	0.5731 (0.4032-0.7500)	0.5576 (0.3818-0.7309)	-0.0183 (-0.02596-0.2543)
***h3***	0.00108 (0.0006-0.0016)	0.0013 (0.0007-0.0019)	0.6450 (0.3241-1.0006)	0.6332 (0.2918-0.9781)	-0.0125 (-0.2216-0.2089)
***28S***	0.0006 (0.0003-0.0008)	0.0011 (0.0005-0.0019)	1.2490 (0.8895-1.6582)	1.4518 (0.9805-1.9531)	0.1892 (-0.0572-0.4451)

Estimations obtained under uncorrelated lognormal relaxed clock. Lower and upper bounds of the 95% HPD are indicated between brackets. Mean Rate: number of substitutions per site across the tree divided by the tree length. Ucld.mean: mean of the branches rates.

**Figure 4:**
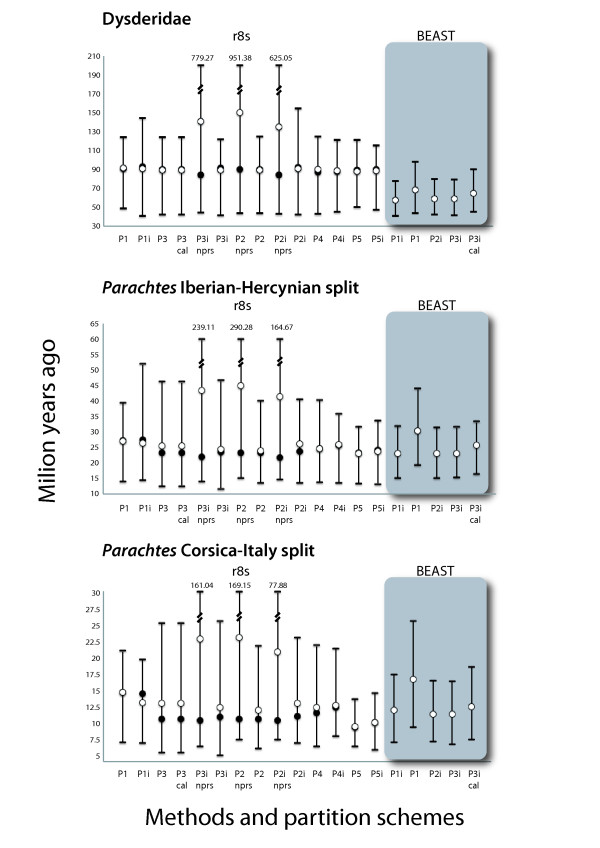
**Estimated age of three nodes selected to monitor the effect of alternative methods and parameters**. Plot of the estimated ages and confidence intervals for three selected nodes recovered under alternative inference methods and partition schemes. Open circles correspond to mean estimated ages obtained from 100 bootstrapped branch lengths obtained with r8s or from the posterior distribution in BEAST; closed circles correspond to estimated ages obtained with r8s. Error bars represent the maximum and minimum values obtained from 100 bootstrapped branch lengths with r8s or the upper and lower bound of the 95% highest posterior density interval obtained with BEAST. Partition schemes as defined in text. i = models include invariants, otherwise they do not; cal = analyses run removing the opening of the Gibraltar strait as fixed calibration point; nprs = run under nprs clock model, otherwise run under penalized likelihood.

The lineage age estimates obtained in r8s under the preferred method selected by cross-validation analyses for the different partition schemes are summarised in Additional file 5. Differences among divergence time estimation methods and partition strategies at different phylogenetic levels were assessed by monitoring the following clades: the Dysderidae node, the *Parachtes *node, and the Corsican-Italian node. The analyses performed with r8s and BEAST yielded similar estimates for the *Parachtes *and the Corsica-Italian nodes, but differed greatly for the deeper Dysderidae node (see Figure 4).

## Discussion

### Timing of *Parachtes *diversification

Although our discussion and conclusions are based on the time estimates obtained using Bayesian methods, we also investigated estimates based on alternative, widely-used smoothing methods. The results showed that time estimates of *Parachtes *diversification are consistent across methods and data partition schemes. The two time estimation methods, however, greatly differed at older time splits, suggesting that there could be incorrect assumptions under certain analytical conditions. Smoothing methods differ from Bayesian methods, as implemented in BEAST, by the use of fixed topologies and roots and by assuming rate autocorrelation between ancestral and descendant branches [2-4,42]. To date, there has been a limited advance in evaluating the strengths and weaknesses of both methods, because direct comparisons between them are hampered by their numerous differences [3]. Finding out the causes of the reported discrepancies are beyond the scope of this study, but our results recommend exerting caution when trying to estimate ages close to the root.

**Figure 5:**
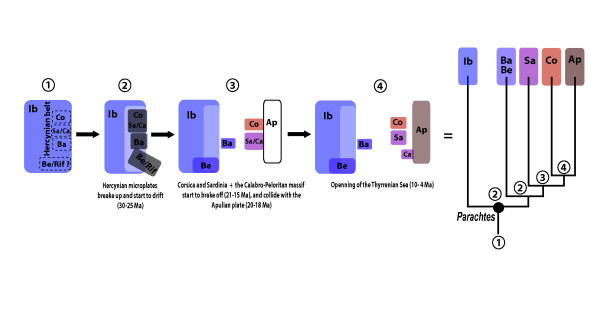
**Reconstruction of the paleobiogeographic scenario for *Parachtes *diversification**. Scenario 1, 2, 3 and 4 represent main geological events that occurred during the opening of the western Mediterranean Basin. Contemporary areas of species distribution are indicated as follows: Ib, Iberian microplate; Co, Corsica; Sa, Sardinia; Ca, Calabria; Ba, Balearic Islands; Be/Rif, Betic/Rift microplate and Ap, Apulian microplate. Current species distribution is indicated at the tips of the inferred topology, and the colours correspond to areas previously defined. The putative geological event involved in the diversification process is indicated at each node.

The tree topology and estimated divergences identify the opening of the Western Mediterranean basin as the main driver for the diversification of *Parachtes *species (Figure 2, 3 and 5). The estimated age for the split of the *Parachtes *stem group closely matches the geochronology of the Hercynian belt break-off, and these node age estimates are robust to alternative analytical procedures and to partition schemes (Figure 4). Therefore, our results reject previous suggestions for a Quaternary origin of the genus [39], mostly based on the absence of these species from the Rift and Kabylies regions in northern Africa. Indeed, the absence of *Parachtes *in northern African exotic terrains poses a challenge to the Miocene origin hypothesis. It may be argued that biotic surveys of the region are far from complete, and that, hence, the presence of the species cannot be completely ruling out. Alternative explanations, such as species extinction due to unfavourable climatic conditions seem unlikely given that other humid fauna with northern Mediterranean affinities, such as the *Salamandra *newts [43] or *Alytes *toads [44], are still found in northern Africa. Evidence of absence is always hard to prove, and further exhaustive, systematic biotic surveys of northwestern Africa will be necessary to discard the presence of *Parachtes *in the region.

Most current evidence indicates that Corsica, Sardinia, northern Sicily, the Balearic Islands, and the Kabylies microplates were part of a single block by the time of the onset of the back arc extension (~30-25 Ma), when the microplates broke and drifted off the Iberian Peninsula [10,12,14,15,28,17]. Nevertheless, the actual location of the Rift-Betic block is still controversial. It has been suggested that the internal part of the Rift-Betic belt was laid near the Balearic Hercynian microplates (see Figure seven of [15]). Some authors go further and propose that the Balearic microplate, together with the Betic belt, formed a Betic-Balearic domain, which suffered a WNW progressive thrust sheet stacking during the back arc extension, ending approximately at the time of the Middle-Miocene (16.4-11.2 Ma) [45,46]. The existence of a Betic-Balearic corridor during the Langhian-Serravallian marine regression (Middle Miocene, c. 14.2 Ma) has been proposed to explain the origin of some Balearic mammalian fossil remains [47]. Alternatively, the Betic-Balearic clade could be the result of a later colonisation facilitated by the land bridge connections between the Balearic Islands and the continent established during the Messinian Salinity Crisis [21,48]. However, our much older time estimates disagree with this scenario.

The sister group relationship of the Sardinian lineage with the remaining Thyrrenian and the Italian species together with the divergence time of these two lineages (14.34 Ma, 20.81-9 Ma) are consistent with the time frame of the separation of Corsica from Sardinia, dated at 21-15 Ma [12]. A similar phylogenetic pattern, i.e., Sardinia as a sister group of Corsica and Italy, has also been reported in the subterranean aquatic stenasellid isopods [20] and the terrestrial isopod *Helleria brevicornis *[32], although in the latter case time estimates suggest an Early Pleistocene split of Corsica and Sardinia.

The close relationships between Corsican and northern Italian species revealed in *Parachtes *have also been recovered in a diverse assemblage of arthropods, including cave crickets, wasps or isopods [20,49-51,32]. In most cases, these links originated as a result of active dispersal during the Pleistocene glacial cycles (2-0.5 Ma), when recurrent marine regressions led to the formation of land bridges between Corsica and Tuscany or when the Tuscany archipelago could have been used as stepping-stones. Our results, however, point towards a deeper split between the Corsican and Italian lineages (11.27, 16.26-6.68 Ma), which matches the onset of the opening of the Tyrrhenian Sea. About 10-9 Ma, Corsica, Sardinia and the Calabro-Peloritan massif separated from the Apennines, after having progressively collided with the Apulian plate at around 20-18 Ma [12,28].

The basal split of Italian *P. andreinii *was estimated at about 9 Ma. Similar divergence times were found between northern and southern Italian species of the *Salamandrina *spectacled salamanders [52]. However, the finding of Miocene-age *Salamandrina *fossils in Sardinia led some authors to propose a double origin for the Italian *Salamandrina*: the ancestor of the northern species would have colonised the Peninsula from Corsica/Sardinia, while the southern one would have drifted eastward from Sardinia on the Calabro-Peloritan massif. The double-origin hypothesis put forward for *Salamandrina *does not match the topology inferred for *Parachtes*. Italian *Parachtes *are monophyletic, and thus a double colonisation scenario would require extinction events on both the islands and the continent. Indeed, the lack of direct links between the Sardinian and any Italian lineage seems to be at odds with geological expectations, given that Sardinia was connected to the Apennines until the opening of the Tyrrhenian Sea [28] and given the biogeographic links such as those reported in cave salamanders of the genus *Hydromantes *[53] and *Discoglossus *toads [21].

With the exception of *P. andreinii*, the estimated timeframe suggests that the diversification of *Parachtes *in the Italian peninsula was mostly driven by Plio-Pleistocene glacial cycles, as already predicted by Alicata [39]. The key role of geographic isolation due to Quaternary climatic oscillations seems to explain the origin of other components of the Italian fauna, such as frogs and snakes [54-58]. Further comprehensive phylogeographical studies on Italian and Iberian *Parachtes *species are, however, needed to gain detailed insight into their evolutionary history and to fully expose the factors responsible for their diversification.

Recent studies have suggested that divergence time estimates under recently developed multilocus coalescent methods may provide younger time estimates than the standard concatenated approach conducted in the present study (see [59]). Although, this issue is still little explored in the literature, the amount of disagreement seems to be particularly significant at shallow divergence times. In fact, it has been argued that the younger estimates obtained with coalescent approaches may be the results of unaccounted recent gene flow [59], since most multilocus coalescent methods of species trees inference assume incomplete lineage sorting as the single source of gene tree incongruence (e.g., [60-62]).

### Estimates of substitution rates

In the absence of fossil or biogeographic calibration points, the extrapolation of substitution rates from independent analyses provides the only information for inferring absolute timeframes. The confirmation that tectonic evolution of the Western Mediterranean shaped *Parachtes *diversification opens the possibility of using this model system to characterise substitution rates in molecular markers of interest.

The use of extrapolated substitution rates for one lineage and markers to date another may be compromised by the variation in rate of molecular evolution among lineages [63]. In the present study, the estimated average substitution rate for the mitochondrial genes estimated (2.25% My^-1^) closely matches the "standard" arthropod mitochondrial DNA clock estimated at 2.3% My^-1 ^[64], one of the most widely used universal rates. These results are surprising, given that the analytical procedures and assumptions used to estimate rates widely differ in the two studies. Nevertheless, Papadopoulou et al. [65] have reached similar conclusions by using another well-dated geological event, the formation of the Mid-Aegean Trench on the eastern Mediterranean, dated at 9-12 Ma, (from 2.39 to 2.69% My^-1^, 3.54% and 1.06%, for mtDNA, *cox1 *and *16S*, respectively). These results may be viewed as a further support for the geological scenario proposed for *Parachtes *diversification.

The few spider rates available in the literature are significantly higher than the ones obtained here. Bond and collaborators [66] estimated a substitution rate for *16S *of 4% My^-1 ^in the trapdoor spider *Aptostichus simus*, while phylogeographic studies in woodlouse hunter spiders endemic to the Canary Island have reported *cox1 *rates as high as 9.8% My^-1 ^[67] or 10.2% My^-1 ^[68]. Interestingly, the former rates were derived from population-level comparisons, which have been shown to produce higher estimates of mutation rates as compared to substitution rates inferred in phylogenetic (species-level) studies (Ho et al. [69]; however, see Emerson [7]). The slower estimates found in *cox1 *(1.75% My^-1^) in a species-level phylogeny of eastern Canarian *Dysdera *[70] supports this last contention.

Our study provides valuable information on the substitution rates of the handful of markers commonly used to infer phylogenies in non-model arthropods. Next-generation sequencing approaches promise to expand the range of molecular markers amenable to phylogenetic inference for organisms such as spiders. Until then, however, rigorous and thorough sampling of phylogeny of non-model organisms may rely on the set of genes used in the present study. In this context, our estimates could be an alternative to obtain absolute time estimates in the absence of calibration points when using such standard sets of molecular markers. Additionally, *Parachtes *may serve as a model system to gain further insights into the rate of molecular, morphological, and ecological trait evolution through time.

## Conclusions

The pattern and timing of species formation in the spider genus *Parachtes *match the geochronological sequence of the opening of the western Mediterranean Basin. Therefore, *Parachtes *spiders provide one of the first and unequivocal pieces of evidence for endemic terrestrial taxa whose origins can be traced back to the separation of the major western Mediterranean islands from the continent (Figure 5). Our results confirm that early Oligocene tectonics played a key role in generating the diversity of the Earth's most populated biodiversity hot-spot [34,71].

## Methods

### Taxonomic sampling

A total of 49 specimens were analysed in the present study (Additional file 1). We included 9 of the 12 currently known *Parachtes *species, along with two new species awaiting formal description: one from Majorca and one from the Italian Peninsula (Figure 1 (B)). The missing species included the Iberian *P. cantabrorum*, the Corsican *P. inaequipes *and the Italian *P. latialis. P. cantabrorum *from an undetermined locality in the Pyrenees [72] has not been collected since its original description (some females assigned to *P. cantabrorum *by Denis are most likely a misidentification, [73]). *P. inaequipes *is known from a single locality in southern Corsica (Bonifacio). One of the specimens included in the present analysis is an unidentified juvenile collected near the *P. inaequipes *type locality (Porto Vecchio, 23 km). Finally, *P. latialis *is known from the Italian district of Lazio. After visiting this area, all collected specimens turned out to constitute a new species.

Half of the *Parachtes *species were represented by single individuals, two by specimens sampled from 2 populations, 2 from 3 populations and one, *P. teruelis*, from 6 populations (Figure 1(B)). We sampled 14 additional species from 5 genera of the same subfamily (Dysderinae) and two genera of the subfamily Harpacteinae, which is also in the family Dysderidae. The genus *Segestria*, a member of the family Segestriidae closely related to Dysderidae and part of the same superfamily Dysderoidea, was included as an outgroup to root trees. The length of the most basal branch in a rooted tree with molecular clock not enforced is trivial and therefore has to be removed before estimating the divergence times using smoothing methods (see below). The most recent common ancestor of Dysderoids, however, provided a calibration point and, therefore, a specimen belonging to Caponiidae, the putative sister-family to Dysderoidea [74], was included to root analyses and preserve the Dysderoid node.

Most specimens were collected in the field by the authors; some were kindly provided by colleagues. Specimens were preserved in 95% ethanol and stored at -20°C in the Department of Animal Biology of the University of Barcelona. Some specimens had been preserved in suboptimal 70% ethanol, but extraction yielded reasonable amounts of DNA for specimens collected within the past 10 years.

### DNA extraction, PCR amplification and sequencing

Genomic DNA was extracted from specimens using the DNeasy Tissue Kit (Qiagen) following the manufacturer's guidelines. Partial fragments of the mitochondrial genes cytochrome c oxidase subunit I (*cox1*), the 12S rRNA (*12S*), a fragment spanning the 3' half of the 16S rRNA ribosomal subunit (*16S *), the complete tRNA leu (*L1*) and the 5' half of the NADH deshydrogenase subunit I (*nad1*), and the nuclear genes 28S rRNA (*28S*), 18S rRNA (*18S*) and Histone H3 (*h3*), were amplified using the following primer pairs: **[*cox1*] **C1-J-1490 [75] and C1-N-2776 [76], alternatively as two overlapping fragments using primer pairs C1-J-1490 or C1-J-1718 [77] with C1-N-2198 [75] or C1-N-2191 [77] and CI-J-2183 [77] with C1-N-2776; **[*16S, L1, nad1*] **LR-N-13398 [77] and N1-J-12350 (Crates: 5'-CCTARTTGRCTARARTTRGCRSATCARCCAATTG-3') or N1-J-12373 [70], or as two overlapping fragments using primers pairs LR-N-13398 with LR-J-12864 [40] and LR-N-12945 [78] with either N1-J-12350 or N1-J-12373; **[*12S*] **12SR-J-14199 and 12SR-N-14594 [79] or 12SR-J-14215 (Viera: 5'-AGGGTGACGGGCGATATG TGCAC-3') and 12SR-N-14522 (Forlan: 5'-AAATTATATACTTTGGCGGC-3'); **[*28S*] **28S-B [80] and 28S-O [76], or as two overlapping fragments using primers pairs 28S-A with 28S-B [80] and 28S-O with 28S-C [76]; **[*18S*] **5F and 9R [80]; **[*h3*] **H3a F and H3a R [81]. PCR conditions were as follows: 2 min. at 94°C followed by 35 cycles of denaturation at 94°C for 30 s, annealing at 42-52°C for 35-45 s (depending on the primers, see below), and extension at 72°C for 30-60 s (depending on the length of the fragment), with a final single extension step at 72°C for 5 min. For the *cox1*, *16S*-*nad1 *and *12S *gene fragments, a successful amplification was achieved with an annealing temperature of 42°C or 45°C for 45 s. For the *18S *and *28S*, a single annealing temperature of 52°C for 35 s was optimum. Finally, *h3 *was amplified using a "touchdown" strategy consistent in beginning the annealing at 60°C for 35 s and lowering proportionally the temperature 1°C in each cycle (during 19 cycles), until reaching a constant temperature of 42°C, keeping this annealing temperature during the following 16 cycles. Amplifications were carried out in a 25 μl reaction volume for a final concentration of 1.25 U *Taq *polymerase (Promega), 2.5 mM MgCl_2 _(Promega), 0.2 mM of each dNTP, 0.2 μM of each primer and about 2 μl of DNA sample and the amount of *Taq *buffer recommended by the manufacturer. PCR products were purified using MultiScreen PCR μ96 cleanup filter plates from Millipore. PCR products were cycle-sequenced in both directions using one of the PCR primers and the BigDye Terminator v3.1 Cycle Sequencing Kit (Applied Biosystem) and sequenced in an ABI 3700 automated sequencer at the Scientific and Technical Services of the University of Barcelona [82]. DNA sequences were edited using Geneious v.5.0.3 [83].

### Phylogenetic Analyses

The alignment of the *cox1*, *nad1 *and *h3 *gene sequences was trivial because there was no evidence for insertion/deletion events and sequences were adjusted manually. Conversely, *12S*, *16S-L1*, *18S *and *28S *sequences showed length polymorphism, and gaps had to be included to retain positional homology. Sequence alignments of each variable length gene were constructed using the online version of the automatic alignment program MAFFT v. 6 [84,85]. The alignment was constructed using the manual strategy option set to Q-INS-i, with default options (Gap opening penalty (GOP) = 1.53 and Offset value, which works like a gap extension penalty (GEP) = 0.0). Two additional alignments were constructed to explore the sensitivity of the phylogenetic results to alternative alignment parameter values. The parameter options were set as follows: GOP = 3 and GEP = 0, to obtain a "compressed alignment", and GOP = 1 and GEP = 0.5, to obtain a "gappy alignment". Gaps were recoded as separate presence/absence characters following Simmons & Ochoterena [86]. Treating gaps in this manner allows the incorporation of gap information in the analyses while minimising the effect of increasing the weight of overlapping multiple non-homologous gaps that result from scoring gaps as an additional state [87]. In addition, absence/presence gap scoring is amenable to Bayesian inference analyses. The program GapCoder [88] was used to facilitate the automatic recoding of the alignments based on the simple method proposed by Simmons et al. [89]. Gene matrices were concatenated using WINCLADA v.1.00.08 [90]. Non-sequenced fragments were scored as missing data.

Concatenation of genes evolving at different rates to resolve phylogenetic relationships among taxa across a wide range of divergence times has been the paradigm in phylogenetic inference for the last two decades. The use of concatenation is based upon the assumption that individual gene trees are congruent among themselves and to the species tree. However, it is well known that different genes may support incongruent topologies due to processes such as horizontal gene transfer, hybridization, gene duplication or incomplete lineage sorting [91-94]. This last problem is especially pervasive when dealing with closely related species, and has recently received a great deal of theoretical and methodological attention. A whole new generation of multispecies coalescent inference methods have been developed to deal with the effects of the stochasticity of the genealogical process. In the present study, however, we have decided to use a concatenation approach based on two main considerations: (1) we did not observe any instance of topological incongruence among individual gene trees, which lead us to assume that none processes know to cause gene incongruence had a major impact on our data, and (2) that our time window of interest (Oligocene/Miocene divergences) minimized the effect of coalescent stochastic errors (i.e. incomplete lineage sorting) in the sampled markers. Moreover, from a practical standpoint, the use of multiple coalescent approaches to infer species trees in the present study would have resulted in losing power to infer topology and branch lengths, given the taxonomic sampling (some species were represented by single individuals), and the low variability of the nuclear markers employed (slow evolving *18S*, *28S *and *h3 *exon).

Parsimony analyses of the individual genes and concatenated matrices were conducted with the program TNT v. 1.0 [95]. Each heuristic search consisted of 1000 iterations of Wagner trees constructed with the random addition of taxa and subsequent TBR branch swapping, holding five trees per iteration and culminating in a final round of branch swapping holding up to 10000 trees. When the number of replicates finding optimal trees was less than 10%, the number of replicates was increased by 1000. Clade support was assessed via jackknife resampling [96] using 1000 replicates with individual heuristic searches consisting of 20 iterations of Wagner tree construction using the random addition of taxa, holding 5 trees per iteration up to 10000 trees.

Bayesian inference analyses were conducted with MRBAYES v.3.1.2 [97] and were run remotely at the Bioportal computer resources of the University of Oslo [98]. The combined matrix was analysed under three scheme partitions: by gene (P1), by gene and mitochondrial protein coding genes 1^st ^+ 2^nd ^positions vs. 3^rd ^positions (P2), and by gene and protein coding genes (both mitochondrial and nuclear) 1^st ^+ 2^nd ^positions vs. 3^rd ^positions (P3). Nucleotide substitution models selected by the Akaike information criterion (AIC) [99], as implemented in jMODELTEST v.0.1.1 [100,101], were specified for each partition, and a standard discrete model was defined for the gaps scored as absence/presence data [102]. The substitution parameters were allowed to vary independently between each partition. Two independent runs of 10 million generations, sampling each 1000 generations, with six simultaneous MCMC (Markov Chain Monte Carlo) chains, each starting from random trees, were carried out simultaneously. The program TRACER v. 1.5 [103] was used to ensure that the Markov chains had reached stationarity by examining the effective sample size (ESS) values and also to determine the correct number of generations to discard as a *burn-in *for the analysis (first 10%). Chain convergence was monitored by ensuring the standard deviation of the split frequencies of these two runs dropped below 0.01.

Maximum likelihood analyses of the concatenated data matrix were conducted with the software program RAxML v. 7.0.4 [104] and run remotely at the CIPRES portal [105]. The same three partition schemes described above were analysed with independent GTR+G substitution models for each partition. The best likelihood tree was selected out of 100 iterations of the random addition of taxa. Non-parametric bootstrap support values were drawn from 100 resampled matrices. Finally, confidence values were mapped onto the best topology.

Trees were visualised and manipulated with the program FigTree v. 1.1.2 [106].

The Approximately Unbiased (AU) topology test [107] implemented in the computer program CONSEL v.0.1i [108] was used to investigate whether alternative topologies could be statistically distinguished (see Results).

### Estimation of divergence times and substitution rates

The use of multiple calibration points results in better and more reliable estimations, while minimising associated uncertainty [109,110]. Absolute ages were thus estimated by incorporating both fossil and biogeographic calibration points, which provided minimum and fixed or maximum ages, respectively. The oldest fossil of the family Segestriidae has been found in Lebanese amber from the Lower Cretaceous [111]. Therefore, the minimum time of separation for the families Segestriidae and Dysderidae was set at 125 Mya. The Harpacteinae fossil genus *Dasumiana *Wunderlich, 2004 from Eocene Baltic amber [111] shows close morphological affinities in the male bulb with the present day genus *Holissus *(Arnedo, pers. obs.). This information was incorporated into the analysis by placing the minimum age estimate of the common ancestor of *Harpactea *and *Holissus *at 35 Mya. The closely-related genus *Dysdera *provided several biogeographic constraints. The split of the Iberian and Moroccan populations of *Dysdera inermis *was assumed to have been caused by the opening of the Strait of Gibraltar and therefore their time of divergence was set at 5.3 Mya. The time of a volcanic island formation provides a maximum age for the lineages inhabiting the island [112]. *Dysdera *has undergone local diversification in the volcanic archipelago of the Canary Islands, and inter-island populations of two endemic Canarian *Dysdera *species were used as additional biogeographic calibration points. The age of emergence of La Palma (2 Mya) and El Hierro (1.2 Mya) [113] placed maximum age estimates for the divergence time between the populations of La Palma and La Gomera of the species *D. calderensis *and for the divergence of the populations of El Hierro and La Gomera of the species *D. gomerensis*.

Minimum and maximum constraints were included as uniform prior distributions to estimate absolute time in a Bayesian framework (see below). Uniform prior distributions for fossil calibration were preferred over more explicit distributions (e.g., lognormal) as a conservative approach, given the limited information available to decide upon the shape of the distribution [114]. For island calibration points, upper bounds were set to the island age and lower bounds to zero. For fossils, the lower bound was set to the latest fossil age and the upper bound was set to 392 Mya, which is the age of the oldest Uraraneida, the putative sister taxon to spiders [115]. The opening of the Gibraltar strait was set as a fixed calibration point following a normal distribution, with mean = 5.3 and sd = 0.15. The effect of using a fixed constraint on time estimates was explored by running additional analyses without this constraint.

Lineage ages were estimated using a Bayesian framework as implemented in BEAST v1.5.4 [116,103] and using multiple rate methods as implemented in r8s v. 1.71 [117]. Species were represented by single specimens (Additional file 1) to avoid very short or zero-length branches, which may negatively influence the performance of the algorithms in r8s [117], and to ensure that only speciation processes as tree prior were included in BEAST estimations.

A preliminary cross-validation analysis was conducted to select the best clock method and, if required, the best smoothing parameter value [118] in r8s analyses. Analyses were conducted using the node constraints described above. Branch lengths were re-estimated with RAxML after taxon removal, enforcing the preferred topology under the alternative partitions schemes described above (P1, P2 and P3) and two additional schemes: gene partition with 3^rd ^codon positions removed (P4) and gene partitions with mitochondrial 3^rd ^codon positions removed (P5). Some concerns have been cast on the simultaneous use of invariants and gamma distributions in evolutionary models [119]. Therefore, branch lengths were reestimated under models with and without invariants for each partition model. Confidence intervals for the time estimates were constructed by generating 100 trees with identical topology by bootstrapping branch lengths, using RAxML.

We used an iterative strategy to select the best clock, partition and speciation model for conducting analyses in BEAST. Alternative schemes were compared using Bayes Factors, as calculated by the program TRACER v1.5. First, lineage ages were estimated under the strict and the two relaxed uncorrelated clocks (exponential and lognormal) using the partition scheme P1 (see above) and selecting the Yule speciation process as tree prior. Once the best clock model was identified, lineage ages were reestimated using the best clock and partition scheme P1, but selecting the Birth-death speciation process as a tree prior. Finally, lineage ages were estimated again by selecting the best clock and speciation process prior, under the three alternative partition schemes (P1, P2, and P3, see above). The best substitution model for each partition was assessed by AIC as implemented in jMODELTEST. An additional run was conducted without considering invariants in the nucleotide substitution models (P1_NOINV_) under the partition scheme P1. Substitution rates were estimated for each gene under the uncorrelated lognormal relaxed clock, the Yule speciation process and the fixed topology in BEAST.

The ultrametric trees estimated with r8s were used as starting trees in BEAST analyses to ensure that time constraints were not violated. Two independent runs of 100 million generations, sampling every 10000 generations, were performed for each analysis. The convergence and mixing of each MCMC chain was assessed with TRACER. Both independent runs of each analysis were combined with LogCombiner after a 10% burn-in, and TreeAnnotator was used to summarise the information from the sampled trees.

## Authors' contributions

MA conceived the idea of the study, and MA and LBB designed the study. Specimens were collected by MA and LBB or kindly provided by colleagues. LBB did the molecular work. LBB and MA conducted the analyses. LBB wrote the first draft, and MA and LBB improved successive versions. Both authors read and approved the final manuscript.

## Supplementary Material

Additional file 1 **Specimens and gene sequence information**. Specimens and sequence accession numbers included in the study with details of sample codes, voucher number, sex (f: female, m: male, juv.: juvenile), and collection locality. Species names with asterisks were included in lineage age estimation analyses. † sequences obtained from different specimens to complete gene sampling nr: near. Click here for file

Additional file 2 **Length of the non-protein coding genes under different alignments**. Default, compressed and gappy alignment lengths are indicated for each gene, along with numbers of gaps and informative gaps codified as absence/presence. Click here for file

Additional file 3 **Substitution models per gene and partitions selected by AIC**. Nucleotide substitution models selected by AIC for a 48 taxon matrix used for phylogenetic analyses, and a 34 taxon matrix under different partitions used for lineage age estimations (see Material and Methods for details). Click here for file

Additional file 4 **Bayes factor comparisons for the selection of the clock model, the speciation process and the partition scheme**. a) Bayes factor comparisons for selection between Yule and Birth-death speciation models under partition scheme P1. b) Bayes factor comparisons for selection among partition schemes P1, P2, P3 and P1_NOINV _(see Material and Methods for details). HME: Harmonic mean of the likelihood from the posterior distribution value. Click here for file

Additional file 5 **Estimated age of three nodes selected to monitor the effect of alternative methods and parameters**. Ages of three selected nodes under different evolutionary models and partition schemes as estimated by R8S, using the best method selected by cross-validation, and BEAST. In R8S analyses, branch lengths were first estimated with RAxML under a fixed topology in Figure 2 and GTR+Γ models, with (INV) or without invariants (NOINV). In BEAST, the best model was selected by jMODELTEST and, for P1 also without invariants (P1_NOINV_). Partition schemes: by gene (P1, 4412 chars), by gene and mitochondrial protein coding genes 1^st ^+ 2^nd ^positions vs. 3^rd ^positions (P2, 4412 chars), by gene and protein coding genes (both mitochondrial and nuclear) 1^st ^+ 2^nd ^positions vs. 3^rd ^positions (P3, 4412 chars), by gene with 3^rd ^codon positions removed (P4, 3762 chars) and by gene with mitochondrial 3^rd ^codon positions removed (P5, 3871 chars). NPRS (Non Parametric Rate Smoothing), PL (Penalized Likelihood), log (logarithmic penalty function). S: Smoothing factor in PL. Age: estimated age. Confidence interval obtained by profiling across 100 trees of constrained topology and bootstrapped branch lengths, as estimated with RAxML: max (maximum value observed), min (minimum value observed), mean (average). 95%HPD: upper and lower bound of the 95% highest posterior density interval. †Analyses run removing the opening of the Gibraltar strait as fixed calibration point. *Preliminary cross validation analyses selected PL log as best fit but failed check. **Analyses under PL in partitions and models where cross-validation selected NPRS as best method, but obtained confidence intervals where absurdly large. Click here for file
