# Supplementary material for: Gone with the plate: the opening of the Western Mediterranean basin drove the diversification of ground-dweller spiders
Source: BMC Evol Biol. 2011 Oct 31;11:317. doi: 10.1186/1471-2148-11-317 (PMC3273451; doi:10.1186/1471-2148-11-317)
Supplement: Additional file 1 — Specimens and gene sequence information. Specimens and sequence accession numbers included in the study with details of sample codes, voucher number, sex (f: female, m: male, juv.: juvenile), and collection locality. Species names with asterisks were included in lineage age estimation analyses. † sequences obtained from different specimens to complete gene sampling nr: near. [file 1471-2148-11-317-S1.PDF]

Additional file 1 - Specimens and genes sequence information

Specimens and sequence accession numbers included in the study with details of sample codes, voucher number, sex (f: female, m: male, juv.: juvenile), and collection locality. Species names with asterisks were included in lineage age estimation analyses.

† sequences obtained from different specimens to complete gene sampling

nr: near

Species	Sample Code	Vouchures	Sex	Locality	*cox1*	*16S-L1*	*nad1*	*12S*	*h3*	*18S*	*28S*
**Caponiidae**											
Caponia sp.	-	ARASP000031	juv.	South Africa, KwaZulu-Natal, St. Lucia Game Reserve, Faines Island	JN689215	JN689214	-	JN689216	-	-	JN705753
**Segestriidae**											
Segestria sp.*	K200	crba000744	f	Iberian Peninsula, Catalonia, Barcelona, Montseny	JN689168	JN705789	JN705789	JN689126	JN689205	JN689079	JN689044
**Dysderidae**											
**Harpacteinae**											
Harpactea fageli *	K247	nhm000041	m	Iberian Peninsula, Madrid, Monasterio de El Paular	JN689144	JN705788	JN705788	JN689104	JN689182	JN689051	JN689040
Holissus unciger *	K480	crba001295	m	Corsica, Pt. du Vecchio, nr. Vivario	JN689149	JN705787	JN705787	JN689107	JN689186	JN689047	JN689036
**Dysderinae**											
Cryptoparachtes sp. *	K484	crba001321	m	Turkey, Ayder	JN689136	JN705754	JN705754	JN689092	JN689178	JN689045	JN689008
Dysdera adriatica *	K450	crba001167		Slovenia, Hrpelje-Kozina, Materija	EU068026/ JN689137	EU068064	EU068064	-	GQ285620	JN689048	GQ28561[illegible]
Dysdera calderensis *	K103	UB4013	m	Canary Islands, La Palma, Garafía, Juan Adalid	AF244309/ JN689138	JN705793	EU139665	JN689093	EU139718	JN689080	EU13978[illegible]
Dysdera calderensis *	L130	crba001454	m	Canary Islands, La Gomera, Monte de La Zarza	JN689139	JN705755	JN705755	JN689094	JN689179†	JN689069	JN689042
Dysdera cf. inermis *	K226	nhm000255	f	Morocco, Tangier-Tétouan, Tangier	EF458142/ JN689141	EF458092	EF458092	JN689100	EU139726	JN689084	EU13979[illegible]
Dysdera crocata *	K418	crba000851	m	Iberian Peninsula, Guadalajara, Hoz de Peregrina	EF458137	JN705758	JN705758	JN689095	GQ285621	JN689070	GQ28561[illegible]
Dysdera gomerensis *	L132	crba001393	f	Canary Islands, La Gomera, Cañada de Jorge	HQ396326	HQ396284	HQ396284	JN689097	HQ396305	JN689081	HQ39631[illegible]
Dysdera gomerensis *	L133	crba001395	m	Canary Islands, El Hierro, Casa Foresta de Frontera	HQ396327	HQ396285	HQ396285	JN689098	HQ396306	JN689085	HQ39631[illegible]
Dysdera inermis *	K228	nhm000075	f	Iberian Peninsula, Andalucía, Cadiz, Tarifa	EF458141/ JN689140	EF458091	EF458091	JN689099	HQ407382	JN689077	HQ40738[illegible]
Dysdera lantosquensis *	K334	crba000678	f	Italy, Tuscany, Monte Argentario	GQ285631	GQ285607/ JN705757	JN705757	JN689096	GQ285626	JN689078	GQ28561[illegible]

Species	Sample Code	Vouchures	Sex	Locality	*cox1*	*16S-L1*	*nad1*	*12S*	*h3*	*18S*	*28S*
**Dysderinae**											
Dysdera silvatica *	K94	UB4155	m	Canary Islands, La Gomera, Barranco de Juel	AF244273/ JN689142	AF244177/ EU139674	EU139674	JN689101	EU139739	JN689083	EU139808
Dysdera silvatica *	K16	UB4177	m	Canary Islands, La Palma, Garafía, Posta de Machín	AF244274	AF244178/ JN705756	JN705756	-	JN689180	-	-
Dysdera silvatica *	X117	nhm000289	m	Canary Islands, El Hierro, Cueva de Longueras	EU068038	EU068072	EU068072	JN689102†	-	JN689050	JN689043
Dysderocrates egregius *	L107	crba001389	f	Romania, Massú Serbea, Baia de Fier, Muierii Cv.	JN689143	JN705759	JN705759	JN689103	JN689181	JN689049	JN689037
Harpactocrates apennicola *	K350	crba000715	m	Italy, Toscana, Massa Carrara, Passo del Cerreto	JN689145	JN705762	JN705762	JN689105	JN689183	JN689088	JN689009
Harpactocrates radulifer *	K60	nhm000300	m	Iberian Peninsula, La Rioja, Vargañón	AF244235/ JN689146	AF244150/ EU139682	EU139682	JN689106	EU139753	JN689052	EU139821
Harpactocrates sp. *	K306	crba000593	f	Turkey, Dedegol Mountains	JN689147	-	-	-	JN689184	JN689071	JN689041
Harpactocrates troglophilus *	K396	crba000992	juv.	Turkey, Burdur	JN689148	JN705760	JN705760	-	JN689185	JN689046	JN689038
Hygrocrates lycaoniae *	K376	crba000827	juv.	Greece, Rhodes, Petaloudes Valley	JN689150	JN705761	JN705761	JN689108	JN689187	JN689089	JN689039
Parachtes andreinii *	LG10	crbaLB000347	m	Italy, Basilicata, Potenza, nr. Fardella	JN689175	JN705771	JN705771	JN689133	JN689211	JN689062	JN689011
Parachtes deminutus	K520	crba001803	f	Iberian Peninsula, Andalucía, Granada, Sierra Nevada, Corral del Veleta	JN689173	JN705790	-	JN689131	-	JN689068	JN689032
Parachtes deminutus *	K521	crba001805	m	Iberian Peninsula, Andalucía, Granada, Sierra Nevada, Puerto de la Ragua	JN689174	JN705791	JN705792	JN689132	JN689210	JN689061	JN689033
Parachtes ignavus *	K479	crba001288	m	France, Corsica, Ajaccio, Foce di Vizzabona	JN689151	JN705763	JN705763	JN689109	JN689188	JN689054	JN689010
Parachtes limbarae	K475	crba001231	f	Italy, Sardinia, Sassari, Punta Balistreri, Monte Limbara	JN689153	JN705772	JN705772	JN689111	JN689190	JN689056	JN689016
Parachtes limbarae	K476	crba001243	m	Italy, Sardinia, Oristano, S. Leonardo, Mt. Ferru	JN689154	JN705773	JN705773	JN689112	JN689191	JN689086	JN689017
Parachtes limbarae *	L270	crba001638	m	Italy Peninsula, Sardinia, Ogliastra, Lanusei	JN689172	JN705774	JN705774	JN689129	JN689209	JN689076	JN689018
Parachtes loboi *	L106	crba000776	m	Iberian Peninsula, Valencia, Alicante, Concentaina	JN689155	JN705775	JN705775	JN689113	JN689192	JN689064	JN689020
Parachtes loboi	K279	crba000418	f	Iberian Peninsula, Valencia, Alicante, Sierra de Aitana, Port de Tudons	JN689156	JN705776	JN705776	JN689114	JN689193	JN689091	JN689026
Parachtes loboi	L136	crbaLB000002	f	Iberian Peninsula, Valencia, Alicante, Serra d'Aitana, Confrides	JN689157	JN705777	JN705777	JN689115	JN689194	JN689072	JN689021
Parachtes loboi *	L137	crbaLB000019	m	Iberian Peninsula, Castilla- La Mancha, Toledo, Robledo del Buey	JN689167	JN705778	JN705778	JN689125	JN689204	JN689074	JN689025
Parachtes n. sp.	K278	crba000387	f	Balearic Islands, Mallorca, Lluc	JN689159	JN705785	JN705785	JN689117	JN689196	JN689058	JN689030
Parachtes n. sp. *	L105	crba001352	f	Balearic Islands, Mallorca, Lluc	JN689160	JN705786	JN705786	JN689118	JN689197	JN689059	JN689031
Parachtes n. sp.	L104	crba001118	m	Italy, Lazio, Frosinone, Monte Cairo	JN689152	JN705765	JN705765	JN689110	JN689189	JN689055	JN689014
Parachtes romandiolae	K352	crba000723	m	Italy, Tuscany, Florence, Vallombrosa	JN689158	JN705766	JN705766	JN689116	JN689195	JN689057	JN689035
Parachtes romandiolae *	K368	crba000716	f	Italy, Tuscany, Massa Carrara, Aulla	JN689162	JN705767	JN705767	JN689120	JN689199	JN689060	JN689015
Parachtes siculus *	L264	crba001636	f	Italy, Sicily, Catania	JN689171	JN705768	JN705768	JN689128	JN689208	JN689090	JN689019
Parachtes sp.	K481	crba001307	juv.	France, Corsica, Porto Vecchio, L'Ospedale	JN689161	JN705764	JN705764	JN689119	JN689198	JN689082	JN689034
Parachtes sp.	K277	nhm000273	juv.	Iberian Peninsula, Navarra, Javier	JN689164	JN705780	JN705780	JN689122	JN689201	JN689065	JN689022
Parachtes sp. *	LG4	crbaLB000341	f	Italy, Lazio, Latina, Campodimele	JN689177	JN705770	JN705770	JN689135	JN689213	JN689063	JN689013
Parachtes teruelis	K204	nhm000293	f	Iberian Peninsula, Aragon, Zaragoza, San Martin de Moncayo	JN689163	JN705779	JN705779	JN689121	JN689200	JN689053	JN689027
Parachtes teruelis *	L103	crba000853	f	Iberian Peninsula, Guadalajara, Hoz de Pelegrina	JN689165	JN705781	JN705781	JN689123	JN689202	JN689066	JN689023
Parachtes teruelis	L135	crbaLB000001	f	Iberian Peninsula, Catalonia, La Noguera, Àger	JN689166	JN705782	JN705782	JN689124	JN689203	JN689073	JN689024
Parachtes teruelis	L260	crbaLB000138	f	Iberian Peninsula, Andalucía, Almería, Cortijo de Umbría	JN689169	JN705783	JN705783	JN689127	JN689206	JN689087	JN689029
Parachtes teruelis	L261	crbaLB000139	f	Iberian Peninsula, Andalucía, Granada, Los Olmos	JN689170	JN705784	JN705784	JN689130	JN689207	JN689075	JN689028

Species	Sample Code	Vouchures	Sex	Locality	*cox1*	*16S-L1*	*nad1*	*12S*	*h3*	*18S*	*28S*
**Dysderinae**											
Parachtes vernae *	LG43	crbaLB000377	m	Italy, Emilia-Romagna, Forlì-Cesena, Bagno di Romagna, Passo dei Mandrioli	JN689176	JN705769	JN705769	JN689134	JN689212	JN689067	JN689012
