# Supplementary material for: Gone with the plate: the opening of the Western Mediterranean basin drove the diversification of ground-dweller spiders
Source: BMC Evol Biol. 2011 Oct 31;11:317. doi: 10.1186/1471-2148-11-317 (PMC3273451; doi:10.1186/1471-2148-11-317)
Supplement: Additional file 2 — Length of the non-protein coding genes under different alignments. Default, compressed and gappy alignment lengths are indicated for each gene, along with numbers of gaps and informative gaps codified as absence/presence. [file 1471-2148-11-317-S2.PDF]

Additional file 2 – Length of the non-protein coding genes under different alignments

Default, compressed and gappy alignment length are indicated for each gene, along with numbers of gaps and informative gaps codified as absence/presence.

	Default AL.			Compressed AL.			Gappy AL.		
**gene**	**length**	**gaps**	**inf. gaps**	**length**	**gaps**	**inf. gaps**	**length**	**gaps**	**inf. gaps**
12S	292	29	18	287	28	16	298	26	16
16S-L1	599	79	29	597	66	30	600	80	32
18S	789	4	2	789	4	2	789	4	2
28S	776	16	7	775	16	7	776	16	7
