# Supplementary material for: Gone with the plate: the opening of the Western Mediterranean basin drove the diversification of ground-dweller spiders
Source: BMC Evol Biol. 2011 Oct 31;11:317. doi: 10.1186/1471-2148-11-317 (PMC3273451; doi:10.1186/1471-2148-11-317)
Supplement: Additional file 3 — Substitution models per gene and partitions selected by AIC. Nucleotide substitution models selected by AIC for a 48 taxon matrix used for phylogenetic analyses, and a 34 taxon matrix under different partitions used for lineage age estimations (see Material and Methods for details). [file 1471-2148-11-317-S3.PDF]

Additional file 3 – Substitution models per gene and partitions selected by AIC

Nucleotide substitution models selected by AIC for a 48 taxa matrix used for phylogenetic analyses, and a 34 taxa matrix under different partitions used for lineage age estimations (see Methods for details).

**gene**	**Model (48 taxa)**	**Model (34 taxa)**
cox1	TIM3+I+G	TIM3+I+G
nad1	GTR+I+G	TIM2+I+G
h3	K80+G	HKY+I+G
12S	GTR+I+G	GTR+I+G
16S-L1	TIM2+I+G	TIM2+I+G
18S	TPM2+I	TMP2+I
28S	GTR+I+G	GTR+I+G
cox1 1st and 2nd positions	TVM+I+G	TVM+I+G
cox1 3rd position	TIM1+I+G	TrN+I+G
nad1 1st and 2nd positions	TrN+I+G	TrN+I+G
nad1 3rd position	TIM2+G	TIM1+G
h3 1st and 2nd positions	TrN+G	TIM2+I
h3 3rd position	K80+G	TPM1+G
