# Supplementary material for: Gone with the plate: the opening of the Western Mediterranean basin drove the diversification of ground-dweller spiders
Source: BMC Evol Biol. 2011 Oct 31;11:317. doi: 10.1186/1471-2148-11-317 (PMC3273451; doi:10.1186/1471-2148-11-317)
Supplement: Additional file 4 — Bayes factor comparisons for the selection of the clock model, the speciation process and the partition scheme. a) Bayes factor comparisons for selection between Yule and Birth-death speciation models under partition scheme P1. b) Bayes factor comparisons for selection among partition schemes P1, P2, P3 and P1NOINV (see Material and Methods for details). HME: Harmonic mean of the likelihood from the posterior distribution value. [file 1471-2148-11-317-S4.PDF]

Additional file 4 – Bayes factor comparisons for the selection of the clock model, the speciation process and the partition scheme

a) Bayes factor comparisons for selection among strict and relaxed exponential and lognormal clock models under partition scheme P1: gene partition.

b) Bayes factor comparisons for selection between Yule and Birth-death speciation models under partition scheme P1.

c) Bayes factor comparisons for selection among partition schemes P1, P2, P3 and $P1_{NOINV}$ (see Material and Methods for details).

HME: Harmoic mean of the likelihood from the posterior distribution value.

a)

Yule speciation process	HME	P1 strict	P1 exponential	P1 lognormal
**P1 strict**	-37591.196	-		
**P1 exponential**	-37366.070	225.126	-	
**P1 lognormal**	**-37365.619**	225.577	0.452	-

b)

Lognormal Clock	HME	P1 Yule	P1 Birth-death
**P1 Yule**	-37365.972	-	
**P1 Birth-death**	**-37365.619**	0.353	-

c)

Yule speciation process & Lognormal Clock	HME	$P1_{NOINV}$	P1	P2	P3
$P1_{NOINV}$	-39113.410	-			
**P1**	-37365.619	1747.792	-		
**P2**	-36635.311	2478.099	730.308	-	
**P3**	**-36370.626**	2742.785	994.993	264.686	-
