# Supplementary material for: Gone with the plate: the opening of the Western Mediterranean basin drove the diversification of ground-dweller spiders
Source: BMC Evol Biol. 2011 Oct 31;11:317. doi: 10.1186/1471-2148-11-317 (PMC3273451; doi:10.1186/1471-2148-11-317)
Supplement: Additional file 5 — Estimated age of three nodes selected to monitor the effect of alternative methods and parameters. Ages of three selected nodes under different evolutionary models and partition schemes as estimated by R8S, using the best method selected by cross-validation, and BEAST. In R8S analyses, branch lengths were first estimated with RAxML under a fixed topology in Figure 2 and GTR+Γ models, with (INV) or without invariants (NOINV). In BEAST, the best model was selected by jMODELTEST and, for P1 also without invariants (P1NOINV). Partition schemes: by gene (P1, 4412 chars), by gene and mitochondrial protein coding genes 1st + 2nd positions vs. 3rd positions (P2, 4412 chars), by gene and protein coding genes (both mitochondrial and nuclear) 1st + 2nd positions vs. 3rd positions (P3, 4412 chars), by gene with 3rd codon positions removed (P4, 3762 chars) and by gene with mitochondrial 3rd codon positions removed (P5, 3871 chars). NPRS (Non Parametric Rate Smoothing), PL (Penalized Likelihood), log (logarithmic penalty function). S: Smoothing factor in PL. Age: estimated age. Confidence interval obtained by profiling across 100 trees of constrained topology and bootstrapped branch lengths, as estimated with RAxML: max (maximum value observed), min (minimum value observed), mean (average). 95%HPD: upper and lower bound of the 95% highest posterior density interval. †Analyses run removing the opening of the Gibraltar strait as fixed calibration point. *Preliminary cross validation analyses selected PL log as best fit but failed check. **Analyses under PL in partitions and models where cross-validation selected NPRS as best method, but obtained confidence intervals where absurdly large. [file 1471-2148-11-317-S5.PDF]

Additional file 5 – Estimated age of three nodes selected to monitor the effect of alternative methods and parameters

Ages of three selected nodes under different evolutionary models and partition schemes as estimated by R8S, using the best method selected by cross-validation, and BEAST. In R8S analyses, branch lengths were first estimated with RAxML under a fixed topology in Fig. 2 and GTR+Γ models, with (INV) or without invariants (NOINV). In BEAST, the best model was selected by jMODELTEST and, for P1 also without invariants ($P1_{NOINV}$). Partition schemes: by gene (P1, 4412 chars), by gene and mitochondrial protein coding genes 1st + 2nd positions vs. 3rd positions (P2, 4412 chars), by gene and protein coding genes (both mitochondrial and nuclear) 1st + 2nd positions vs. 3rd positions (P3, 4412 chars), by gene with 3rd codon positions removed (P4, 3762 chars) and by gene with mitochondrial 3rd codon positions removed (P5, 3871 chars). NPRS (Non Parametric Rate Smoothing), PL (Penalized Likelihood), log (logarithmic penalty function). S: Smoothing factor in PL. Age: estimated age. Confidence interval obtained by profiling across 100 trees of constrained topology and bootstrapped branch lengths, as estimated with RAxML: max (maximum value observed), min (minimum value observed), mean (average). 95%HPD: upper and lower bound of the 95% highest posterior density interval. †Analyses run removing the opening of the Gibraltar strait as fixed calibration point. *Preliminary cross validation analyses selected PL log as best fit but failed check. **Analyses under PL in partitions and models where cross-validation selected NPRS as best method, but obtained confidence intervals where absurdly large.

			Dysderidae				*Parachtes* Iberian-Hercynian split				*Parachtes* Corsica-Italy split			
				Confidence interval				Confidence interval				Confidence interval		
**Partition**	**Method**	**S**	**Age**	**max**	**min**	**mean**	**Age**	**max**	**min**	**mean**	**Age**	**max**	**min**	**mean**
$P1_{NOINV}$	R8S PL log	1000	91.19	123.89	48.83	91.52	27.25	39.43	14.10	26.99	14.62	20.95	6.91	14.58
$P1_{INV}$	R8S PL log	1000	93.28	144.16	41.12	90.99	27.60	52.02	14.54	26.34	14.40	19.57	6.84	12.97
$P3_{NOINV}$	R8S PL log	320	90.04	124.07	42.35	89.70	23.39	46.31	12.34	25.49	10.50	25.10	5.42	12.93
$P3_{NOINV}$†	R8S PL log	320	90.04	124.07	42.35	89.70	23.39	46.31	12.34	25.49	10.50	25.10	5.42	12.93
$P3_{INV}$	R8S NPRS	-	84.52	779.27	44.73	140.58	21.92	239.11	13.94	43.39	10.32	161.04	6.30	22.79
$P3_{INV}$	R8S PL log **	32	91.71	121.75	41.38	89.25	23.44	46.84	11.66	24.41	10.78	25.41	4.97	12.25
$P2_{NOINV}$	R8S NPRS*	-	90.04	951.38	43.57	150.02	23.39	290.28	15.12	45.09	10.50	169.15	7.32	22.95
$P2_{NOINV}$	R8S PL log **	3.2	89.93	125.00	43.56	89.74	23.20	40.12	13.63	24.07	10.46	21.68	6.02	11.87
$P2_{INV}$	R8S NPRS*	-	84.63	625.05	42.79	135.07	21.84	164.67	14.60	41.38	10.32	77.88	7.33	20.74
$P2_{INV}$	R8S PL log **	1000	92.05	154.51	42.17	90.62	23.73	40.65	13.60	26.09	10.96	22.98	6.80	12.90
$P4_{NOINV}$	R8S PL log	100	86.97	124.99	43.37	90.16	24.65	40.25	13.71	24.73	11.44	21.76	6.31	12.23
$P4_{INV}$	R8S PL log	320	88.28	121.42	45.17	88.42	25.63	35.99	13.66	25.85	12.25	21.32	7.93	12.58
$P5_{NOINV}$	R8S PL log	100	89.45	121.14	50.09	87.66	23.22	31.76	13.28	23.02	9.21	13.58	6.26	9.33
$P5_{INV}$	R8S PL log	320	90.49	115.47	47.18	88.42	24.11	33.65	13.16	23.80	10.00	14.49	5.74	9.93
				**95%HPD upper**	**95%HPD lower**	**mean**		**95%HPD upper**	**95%HPD lower**	**mean**		**95%HPD upper**	**95%HPD lower**	**mean**
P1	BEAST	-	-	77.85	41.01	57.36	-	32.03	15.02	22.98	-	17.33	6.96	11.90
$P1_{NOINV}$	BEAST	-	-	98.16	43.83	68.20	-	44.12	19.21	30.46	-	25.44	9.25	16.55
P2	BEAST	-	-	79.97	42.56	59.33	-	31.55	15.10	23.09	-	16.40	7.02	11.20
P3	BEAST	-	-	79.59	41.82	59.19	-	31.81	15.32	23.16	-	16.28	6.65	11.27
P3†	BEAST	-	-	90.39	45.21	64.93	-	33.37	16.54	25.69	-	18.41	7.35	12.41
